# Effect of Electron-Beam Radiation and Other Sterilization Techniques on Structural, Mechanical and Microbiological Properties of Thermoplastic Starch Blend

**DOI:** 10.1007/s10924-020-01972-9

**Published:** 2020-11-21

**Authors:** Anna Iuliano, Agata Fabiszewska, Katarzyna Kozik, Magdalena Rzepna, Justyna Ostrowska, Maciej Dębowski, Andrzej Plichta

**Affiliations:** 1 Department of Chemistry, Institute of Food Sciences, Warsaw University of Life Sciences – SGGW, Nowoursynowska 159c, 02-776 Warsaw, Poland; 2 Faculty of Chemistry, Warsaw University of Technology, Noakowskiego 3, 00-664 Warsaw, Poland; 3 Institute of Nuclear Chemistry and Technology, Dorodna 16, 03-195 Warsaw, Poland; 4 Department of Organic Technologies, The Łukasiewicz Research Network – New Chemical Syntheses Institute, al. Tysiąclecia Państwa Polskiego 13A, 24-110 Puławy, Poland

**Keywords:** Electron-beam radiation, Starch, *B. subtilis*, Degradation, Mechanical properties

## Abstract

This work investigates the potential application of various sterilization methods for microorganism inactivation on the thermoplastic starch blend surface. The influence of the e-beam and UV radiation, ethanol, isopropanol and microwave autoclave on structural and packaging properties were studied. All the applied methods were successful in the inactivation of yeast and molds, however only the e-beam radiation was able to remove the bacterial microflora. The FTIR analysis revealed no significant changes in the polymer structure, nevertheless, a deterioration of the mechanical properties of the blend was observed. The least invasive method was the UV radiation which did not affect the mechanical parameters and additionally improved the barrier properties of the tested material. Moreover, it was proved that during the e-beam radiation the chain scission and cross-linking occurred. The non-irradiated and irradiated samples were subjected to the enzymatic degradation studies performed in the presence of amylase. The results indicated that irradiation accelerated the decomposition of material, which was confirmed by the measurements of weight loss, and mass of glucose and starch released to the solution in the course of biodegradation, as well as the FTIR and thermal analysis.

**Electronic supplementary material:**

The online version of this article (10.1007/s10924-020-01972-9) contains supplementary material, which is available to authorized users.

## Introduction

Sterilization is a process, which allows for a complete destruction or removal of all microorganisms that could contaminate packaging materials and thereby constitute a health hazard [1]. The choice of a proper sterilization technique depends on the material used. There is no universal method for polymer sterilization. Every potential material has to go through a detailed analysis of its susceptibility to sterilization and all the packaging properties have to be investigated. Some of the polymers (both natural and synthetic) treated with superheated steam, hot air or high temperature undergo deformation, thermal and oxidative degradation and in many cases hydrolysis if they are susceptible. In those circumstances better results might be provided by other methods such as: ethylene oxide [2], hydrogen peroxide [3], ethyl alcohol [4], gamma [5] and electron-beam (e-beam) radiation [6], plasma [7]. Moreover, the process of choosing the right method is even more complicated, if we look for the sterilization technique dedicated for food packaging. In that case, the analysis concerns the sterilization effect on microbiological interactions, packaging properties and changing biomolecules in food [8].

The most commonly used biodegradable material in the food packaging industry is thermoplastic starch, TPS [9]. TPS can be obtained in the course of destruction of the crystalline phase of the native starch by thermal and mechanical methods using a plasticizer, such as water, glycerol, urea or sorbitol, which reduces the glass transition temperature and melting point of starch [10]. After the modification, starch can be processed by standard methods used for thermoplastic polymers such as extrusion, injection molding, thermoforming and hot-pressing [11]. However, high hydrophilicity, low degradation temperatures and poor mechanical properties of TPS give a limitation for its various industrial applications [12]. The solution to this problem can be blending of starch with more hydrophobic biodegradable polyesters. The most studied starch blends are those based on poly(butylene adipate-*co*-terephthalate), PBAT [13, 14], poly(ε-caprolactone), PCL [15, 16], polylactide, PLA [11] and poly(butylene succinate), PBS [17, 18]. In all cases, the introduction of TPS into polyester matrix caused significant reduction in mechanical properties and several attempts were made in order to improve the miscibility with biodegradable polyesters. For this purpose poly(ethylene-*co*-vinyl alcohol), maleic anhydride [13] as well as PCL [19], PLA [20], PBAT [21], grafted with maleic anhydride and​/or glycidyl methacrylate were used. However, it was also proved that mechanical properties and morphology of the PBAT/TPS blend can be controlled solely by changing the compounding parameters [14].

In the presented paper the influence of various sterilization techniques on the structure and packaging properties of the TPS/PBS blend (50/50 wt%) plasticized with 5 wt% of glycerol and 25 wt% of urea were examined. PBS is a biodegradable polymer with good mechanical and thermal properties, good processability and high chemical resistance. Moreover, it can be obtained from the renewable resources. In the literature, numerous studies regarding blending of TPS with PBS can be found, however so far, none of them reported the susceptibility of such a blend to sterilization. For our studies we selected some standard sterilization techniques such as: the e-beam and UV radiation, a treatment with a isopropanol and ethanol as well as microwave autoclave and we evaluated their sterilization efficiency. Due to the fact, that some of those techniques can change the material properties and its structural characteristic, the susceptibility to degradation as well as thermal, mechanical, morphological, and barrier properties were also investigated.

## Materials and Methods

### Materials

α-Amylase from *Aspergillus oryzae* (powder, ∼ 30 U mg^−1^), sodium azide (> 99.5%), trichloroacetic acid (TCA, 99%), dinitrosalicylic acid (DNS, 98%) were obtained from Sigma-Aldrich (USA). All medium ingredients (agar–agar, yeast extract, peptone, Sabouraud chloramphenicol agar) were purchased from BTL (Poland). Soluble starch (pure p.a.), glucose (pure p.a.), citric acid monohydrate (99.5%), inorganic salts [NH_4_NO_3_ (pure p.a.), (NH_4_)_2_SO_4_ (pure p.a.), K_2_HPO_4_ (pure p.a.), KH_2_PO_4_ (pure p.a.), NaCl (pure p.a.)], isopropanol (PrOH, 99.5%) and ethanol (EtOH, 96%) were obtained from POCH (Poland). All reagents were applied without further purification. Τhe PBS homopolymer (Bionolle 1001MD) was supplied by Showa Denko (Japan). Native potato starch was purchased from “TRZEMESZNO” Sp. z o.o. Potato Industry Company (Poland). Urea was obtained from Grupa Azoty Zakłady Azotowe “Puławy” (Poland) and glycerol (99.5%) purchased from Brenntag (Poland).

### Preparation of the TPS/PBS Blend

The film sample made of the PBS/TPS (50/50 wt%) blend was prepared by the blown film method using a single screw extruder (Labtech Engineering, Thailand, L/D = 30, D = 20 mm). The temperatures ranging from 130 to 140 °C were applied and the die temperature was set at 140 °C. Starch was plasticized by urea and glycerol (25 wt% and 5 wt%, respectively). The neat PBS sample was prepared by the same method, but at the different temperature—the extruder was operated at 135–150 °C. The PBS film was characterized by M_w_ = 78.9 kg mol^−1^ and Ð_M_ = 7.9.

### Sterilization Techniques

#### Irradiation

The TPS/PBS film was irradiated on the one side of the polymer foil in an air atmosphere at ambient temperature with doses of 5, 13 and 26 kGy using 10 MeV electron beam generated in the linear electron accelerator Elektronika 10/10. The UV irradiation (λ = 254 nm) was performed using a 6 W lamp. Each sample was irradiated for 20 min (10 min per each side).

#### Thermal Sterilization in Microwave Autoclave

The polymer foil placed in the 500 mL flask filled with 200 mL of distillated water and sterilized using a MicroJet microwave autoclave (Enbio, Poland) at temperature of 121 °C for 20 min. After the sterilization process, water from the flask was aseptically removed.

#### Alcohol Sterilization

Each sample was incubated in a diluted ethanol (70%) or isopropanol for 30 min and washed with a sterile distilled water.

### Gas Chromatography

A Shimadzu gas chromatograph type GC 2014 equipped with a thermal conductivity detector and column packed with molecular sieves 5A, was applied for analyses of the gaseous products formed in samples submitted for the electron irradiation (hydrogen analysis), as well as for the calculation of the loss of oxygen from the atmosphere surrounding the sample in the irradiation vessel. The chromatographic system was working at 100 °C, whereas the column and detector were kept at 120 °C. The rate of the carrier gas flow was 10 mL min^−1^.

### Structural and Thermal Properties

The FTIR spectra were recorded on a Nicolet iS5 ATR Thermo Scientific spectrometer equipped with a diamond crystal iD7 ATR sampling component. The molar mass and polydispersity (Ð_M_) were determined by gel permeation chromatography (GPC) on a Viscotek system comprising GPCmax and TDA 305 triple detection unit (RI, IV, LS) equipped with one guard and two DVB Jordi gel columns (102–107, linear, mix bed) in CH_2_Cl_2_ as eluent at 35 °C at a flow rate of 1.0 mL min^−1^. The results were analyzed by OMNIC 9.0 software. DSC analysis was carried out with a TA Instrument Model Q20 calorimeter under nitrogen flow. The sample was heated from 20 to 150 °C at a heating rate of 10 °C min^−1^ and cooled from 150 to − 90 °C at the same heating rate. Next, a second heating scan was performed from − 90 to 150 °C at a heating rate of 20 °C min^−1^ to determine T_g_ temperature. The thermal stability between 30 and 600 °C was investigated using a TA Instrument TGA Q500 instrument under a steady flow of nitrogen (60 mL min^−1^) at a heating rate of 10 °C min^−1^.

### Scanning Electron Microscopy

Scanning electron microscopy (SEM) images were obtained on a Zeiss Ultra Plus apparatus working at a voltage of 2 kV. Before the measurements the polymer film was covered with an electron-conductive carbon layer utilizing a high-vacuum sputter coater.

### Mechanical and Barrier Properties

The tensile properties were examined with an Instron mechanical tester, model 5566, at tensile speed of 10 mm min^−1^ at room temperature with head of maximum load of 100 N. The Young’s modulus, tensile strength and elongation at break were measured. The measurements were repeated from four to five times, for each material. Barrier properties were determined based on the water vapor permeability (WVP) according to Nowacka et al. [22]. The barrier measurement was repeated three times for each material.

### Analysis of Microorganisms Inactivation by Different Sterilization Techniques

The TPS/PBS foil with dimension of 3 × 3 cm was sterilized by a selected sterilization technique and then incubated for 14 days in culture media, such as: nutrient agar prepared according to PN-EN ISO 4833:2004 [for determination of the total number of bacteria (OLB)]; medium for determining the total number of yeasts (YPG) and medium for a mold growth (PL) prepared according to PN-ISO 7954:1999. The flask cultures were put on a IKA KS 4000 ic control rotary-shaker at 140 rpm, at 20 °C for the mold cultivation or at 28 °C for the yeast and bacteria growth. The optical density of media incubated with TPS/PBS polymer film was measured each day at a wavelength of 600 nm to estimate the growth of microorganisms which survived the sterilization process. The medium was considered as infected when the OD_600_ value exceeded 0.7 what corresponded to the number of microorganism cells of 6.0–7.0 log cfu mL^−1^. In that case the flask culture was being disturbed and the culture broth was used for identification of the species that originated from sterilized polymer films. The liquid culture media contained: 2.5 g L^−1^ yeast extract, 5 g L^−1^ peptone, 10 g L^−1^ glucose for the OLB medium; 10 g L^−1^ yeast extract, 20 g L^−1^ peptone, 20 g L^−1^ glucose for the YPG medium and 1 g L^− 1^ NH_4_NO_3_, 1 g L^−1^ (NH_4_)_2_SO_4_, 4 g L^−1^ K_2_HPO_4_, 2 g L^−1^ KH_2_PO_4_, 1 g L^−1^ NaCl, 10 g L^−1^ glucose, 1 g L^− 1^ yeast extract, 57 mL L^−1^ citric acid for the PL medium. To prepare solid medium, 20 g L^−1^ of agar was additionally added. The concentration of Sabouraud chloramphenicol agar was 44.5 g L^−1^.

### Isolation and Identification of Microorganism from Polymer Films

The second stage of the experiment concerned the isolation of microorganisms existing on the films after sterilization, which were grown in flask cultures during a 14-day of incubation in culture media. From each infected flask 1 mL of culture broth was inoculated onto agar plates (2% of agar medium in relation to the amount of medium used in a flask culture experiment). In parallel, for OLB culture broth an additional inoculation was performed on Sabouraud agar medium containing chloramphenicol. In such samples the bacterial growth was inhibited, which allowed to determine whether yeast or molds were present in the supernatant. The inoculated plates were incubated for three days at 20 °C or 28 °C. Individual colonies were isolated, kept on agar slants at 4 °C and subjected to species identification.

To identify isolated microorganisms, selected regions of the 16S and 26S rRNA genes were amplified by PCR (polymerase chain reaction) (Applied Biosystems Thermal Cycler) using primers of the highly conserved regions found within these sequences: 27F (5′-AGA GTT TGA TCM TGG CTC AG-3′) and 1492R (5′-CGG TTA CCT TGT TAC GAC TT-3′) for bacteria and NL1 (5′-TGC TGG AGC CAT GGA TC-3′) and NL4 (5′-GGT CCG TGT TTC AAG ACG G-3′) for fungi. The PCR product was sequenced by the Sanger method and the results were assembled in the Geneious software. The obtained DNA sequences were compared with the 16S and 26S rRNA sequences in the NCBI GenBank database, which allowed to identify the cultured microorganisms.

### Cultivation of *B. subtilis* and the Enzyme Activity Measurements

A wild strain of *Bacillus subtilis* with a high amylase activity was selected from the microflora of organisms inhabiting the TPS/PBS films. Enzymes produced by the bacterial strain were used to degrade the TPS/PBS film.

*Bacillus subtilis* for the synthesis of amylolytic enzymes was cultured in a medium of pH 7.5 containing 5 g L^−1^ yeast extract, 10 g L^−1^ peptone, 20 g L^−1^ starch, 10 g L^−1^ NaCl in 500 mL flasks on a rotary shaker at 37 °C. The 24 h culture of the bacterial cells in the OLB medium was used as an inoculum in a volume of 2 mL. The flasks were removed every 24 h and 1 mL of the culture supernatant was centrifuged for 10 min at 10,000 rpm for amylolytic enzyme assay. The measurement of amylase activity was performed according to a modified method described in the literature [23]. 50 µL of starch solution (0.01 g mL^−1^ in phosphate buffer of pH 6.4) and 50 µL of clear supernatant was added to the glass vials. The blank sample, without supernatant, was also prepared. The reaction was carried out for 30 min at 50 °C and stopped by adding 50 µL of TCA. Additionally, 50 µL of supernatant added to the blank sample for volume equalization. Immediately before the absorbance measurement 5 mL redistilled water and 150 µL of iodine were added. The absorbance of the solution was determined at a wavelength of 580 nm. One unit (U) of enzyme activity for the starch–iodine assay was defined as the disappearance of an average of 1 mg of iodine binding starch min^−1^ in the analyzed reaction. The U mL^−1^ value was calculated using the formula: U mL^−1^ = (*A*_580blank_ – *A*_580sample_)/*A*_580/mg starch_ 30 min^−1^ 0.05 mL^−1^, where *A*_580blank_ is the absorbance obtained from the starch without the addition of enzyme, *A*_580sample_ is the absorbance for the starch digested with enzyme, *A*_580/mg starch_ is the absorbance for 1 mg of starch as derived from the standard curve, 30 min is the assay incubation time, and 0.05 mL is the volume of the enzyme used in the assay.

### Degradation in the Presence of Commercial Enzymes and Enzyme of Microbial Origin

The TPS/PBS film were cut into 2.0 × 2.0 cm strips with thickness 0.2 mm. Each film was placed in a separate Erlenmeyer flask containing 10 mL of acetate buffer (pH 5.5), 6 mg of αamylase and 60 mg of sodium azide. In case of degradation in the presence of enzyme from *B. subtilis*, 10 mL of post-culture liquid was placed in an Erlenmeyer flask and 60 mg of sodium azide was added. The film-enzyme incubations were carried out at 37 °C in a rotary shaker (150 rpm). At the specific time points, respective films were removed from the shaker incubator, rinsed thoroughly with distilled water, and then dried under reduced pressure (0.5 mbar) at room temperature for 48 h. The experimental weight loss values represent the averages of weight measurements carried out for the three replicate films. The starch and reducing sugars concentration in the solution resulting from a degradation experiment was determined according to the procedures previously described [24].

### Degradation in the Presence of Wild *B. subtilis* Strain in the Solid Agar Medium

Mineral medium, prepared according to the procedure described in the ASTM G21-96(2002) standard, was aseptically poured into Petri plates. TPS/PBS film was cut into 2 × 2 cm strips, irradiated with a dose of 5 kGy and sterilely placed in the center of Petri plates containing the mineral medium deprived of any carbon source. The inoculum of 0.2 mL was transferred on each sample and smoothly spread using a cell spreader. The petri plates were incubated at 37 °C. After 1, 2 and 3 weeks, respective films were removed, washed with ethyl alcohol, and then dried under a reduced pressure (0.5 mbar) at room temperature for 48 h. The experimental weight loss values represent the averages of weight measurements carried out for the three replicate films. The inoculum from *B. subtilis* was prepared using the OLB medium and the initial spore concentration was 1.81 × 10^8^ cfu mL^−1^.

## Results and Discussion

### Microbiological Purity Test

In order to determine the microorganism inactivation ability of the selected sterilization techniques, the TPS/PBS polymer film was sterilized with each technique and aseptically placed in the bacterial, yeast and mold culture media. The incubation was stopped when the microbial growth was observed and the optical density at 600 nm exceeded 0.6. The obtained results are summarized in Table 1. A 30 min incubation time in alcohols was chosen based on the preliminary experiments, which showed that the OLB medium was infected already after 24 h for the sample incubated for 1 min and 10 min in isopropanol. For this reason we extended the exposure time to 30 min.

**Table 1:** Results of sterilization method of the TPS/PBS blend

Sterilization method	Contact time	Result of sterilization method
Autoclave	20 min	Infected within 1–4 days; isolated species: *B. altitudinis, B. aerophilus* *B. stratosphericus, B. pumilus*
PrOH	30 min	Infected within 3–4 days; isolated species: *B. subtilis, B. gibsonii*
EtOH	30 min	Infected after 2 days; isolated species: *B. subtilis, B. gibsonii*
UV	10 min per side	Infected within 2–3 days; isolated species: *B. subtilis, B. gibsonii*
e-Beam irradiation	Direct exposure: 2–4 s	Effective in inactivating microorganisms^a^

^a^The presented data were determined independently of the irradiation dose used

All of the applied e-beam radiation doses were effective in microorganism inactivation. The radiation dose of 26 kGy kept the microbial population below 1 log_10_ cfu mL^−1^ after 14 days of incubation at 30 °C. The other sterilization techniques were successful in mold inactivation, however the microbial growth was observed between 24 h and 96 h in OLB and YPG medium. In those cases, the streaking was done for each cultivation in order to isolate a single colony and analyze using genetic techniques, the type of species which survived the sterilization conditions. The isolated species are presented in the last column of Table 1. As can be seen, the polymer film surface was inhabited by *Bacillus* genera.

In general, steam and dry heat sterilization are considered as the best sterilization techniques due to the high penetration ability and the absence of toxic residues [25]. The mechanism of sterilization is based on the destruction of replication metabolic and structural components of microorganisms and in case of dry heat also dehydration followed by oxidation [4]. However, according to the literature, *Bacillus* sp. spores are highly resistant to heat and other sterilization techniques. This phenomena is associated with impermeability, low water content, high levels of pyridine-2,6-dicarboxylic acid and divalent cations, and outer membrane thickness [26]. Moreover, the spore DNA is protected against various types of damage. That is why, steam sterilization, alcohol solutions [27] and the UV radiation [28] did not inactivate the *B. subtilis* spores and all those methods have to be supported by other sterilization techniques. In case of ionizing radiation, mechanism of inactivation covers structural damage, spilling of cytoplasmic contents, reduction in membrane integrity and fragmentation of genomic DNA. The e-beam radiation has the ability to inactivate both gram-negative and gram-positive bacteria, however, some endospores are resistant even to high doses of radiation [29, 30] and some additional heat treatment is required for the complete microorganism inactivation. In our previous studies [6] we proved that the e-beam radiation is effective in microorganism inactivation on a hydrophobic surface of the PBAT/PLA polyester blend. According to the current results, it seems that it is also possible to sterilize the hydrophilic surface of the TPS/PBS blend, even by using a very small dose of radiation. It can be concluded that there is not a wide range of methods allowing for a complete microorganisms inactivation. Although the e-beam radiation seemed to be the perfect method for the TPS/PBS blends sterilization, a high cost of this method inclines to further experiments on a combination of radiation and some physicochemical methods for inactivation of the microflora colonizing surfaces of those polymers. However, combined techniques of sterilization will be the subject matter of another article.

### FTIR Characterization of the TPS/PBS Blend Before and After Sterilization

**Fig. 1:**
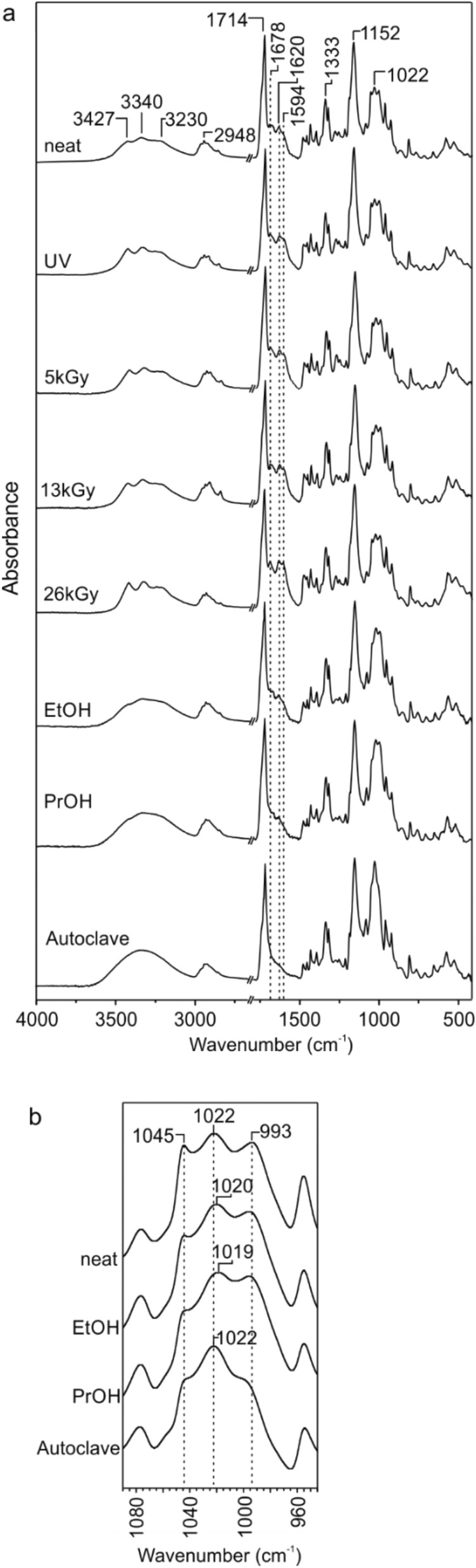
**a** FTIR spectra of the TPS/PBS blends before and after sterilization by: UV irradiation, e-beam irradiation with the doses of 5 kGy, 13 kGy, 26 kGy, ethanol, isopropanol and microwave autoclave, and **b** FTIR spectra (a 1085–950 cm^−1^ region) of the TPS/PBS blend before and after sterilization in alcohol solutions or microwave autoclave

The structure of material before and after sterilization was analyzed based on the FTIR spectra, in which several regions characteristic for starch, PBS, urea and glycerol were observed (Fig. 1a). The O–H and C–O stretching vibrations of the starch give strong bands at the range of 36,003,000 cm^−1^ and 1022–970 cm^−1^, respectively. The first region (3427–3255 cm^−1^) can be also attributed to N–H stretching bands of urea, however, the most characteristic signals for urea are located at 1678 cm^−1^, 1594 cm^−1^, 1460 cm^−1^ assigned to the C=O stretching mode, N–H bending band and C–N stretching vibration, respectively [24]. The FTIR absorption band at 3000–2800 cm^−1^ is attributed to the C–H stretching vibrations in both starch and PBS. In case of PBS the absorption bands located at 1714 cm^−1^ and 1152 cm^−1^ were attributed to the C=O and C–O stretching vibrations in the ester group, respectively [31].

The FTIR spectra of TPS/PBS blend are difficult to analyze due to a high overlap of several bands making their assignment very complicated, however, few conclusions can be drawn. According to the literature data, the UV radiation does not cause significant changes in the FTIR spectrum of starch, even after 13 h of exposure [32]. As can be seen in Fig. 1a, all signals in the analyzed regions, did not change the position or intensity. The same observation was conducted for the polymer films treated with the e-beam radiation. In this case, we would expect some decrease in the intensity of the bands between 3000 and 2800 cm^−1^, as the hydroxyl radicals formed by water radiolysis rapidly attack the C–H bonds liberating the hydrogen atom from the bond [33]. However, due to the fact, that the radiation was performed under non-aqueous conditions such changes were negligible, even if some amount of water, used as a TPS plasticizer, was present in the blend. The sterilization by alcohols and microwave caused partial leaching of plasticizers observed as a decrease in the intensity of the band in the 1690–1550 cm^−1^ region. Additionally, due to the lower content of urea in the blend, one can observe the change in the intensity ratio of the starch signals located between 1022 and 993 cm^−1^, which indicates the weakening of hydrogen bonds between starch and plasticizers (Fig. 1b) [34].

### Thermal Analysis

Differential scanning calorimetry (DSC) was used to identify the transition temperatures and enthalpy of fusion of the PBS phase as well as its degree of crystallization (*x*_c_). All these data are collected in Table 2. As can be seen, all samples exhibited melting and crystallization. Additionally, during the 2nd heating cycle (Fig. S1 in Supplementary Material) a double melting peak was observed which is associated with the melt-recrystallization process of the high molar mass PBS. These two peaks represent two different distributions of lamellae having different thermal properties. This phenomena is well known and described in the literature [35].

**Table 2:** Thermal parameters of the TPS/PBS blend before and after sterilization determined by DSC and TG

Sample	T_g_ (°C)	T_m_ (°C)	T_c_ (°C)	*x*_*c*_ (%)	Mass loss (%) up to 230 °C	Mass loss (%) up to 450 °C	T_onset3_ (°C)	T_onset4_ (°C)	T_onset3*_ (°C)	T_onset4*_ (°C)
Neat	− 30.1	111	88.1	54.7	11.0	78.8	300	374	–	–
UV	− 27.5	112	87.6	56.6	8.5	82.2	302	376	–	–
5 kGy	− 29.3	112	87.6	58.1	10.5	79.8	301	375	299	372
13 kGy	− 29.8	111	87.9	58.4	10.2	79.5	302	375	298	372
26 kGy	− 29.6	112	87.1	62.3	10.4	78.2	302	375	295	370
EtOH	− 30.3	111	87.9	58.4	–	–	–	–	–	–
PrOH	− 29.9	112	88.0	57.0	–	–	–	–	–	–
Autoclave	− 32.9	110	78.1	73.8	–	–	–	–	–	–

T_g_ (glass transition) and T_m_ (melting temperature) were determined by the 2nd and 1st heating scan, respectively. T_c_ (melt crystallization temperature) was determined by cooling scan. The crystallinity degree (*x*_*c*_) was calculated according to equation *x*_c_ = Δ${\text{H}}_{m}/(\Delta{\text{H}}_{m}^{o}\cdot \omega ) \cdot 100,$ where Δ${\text{H}}_{m}^{o}$ = 110.5 J g^−1 31^ and ω is a weight percentage of PBS in the blend. T_onset3_ and T_onset4_ relate to the degradation of TPS and PBS in the polymer blend, respectively. The T_onset_ values of the respective degradation steps estimated for the irradiated samples stored for 26 months are indicated with *

The melting point (T_m_), glass transition (T_g_) and melt crystallization temperature (T_c_) of PBS were almost unaffected by the UV or e-beam radiation, although degree of crystallinity increased with increasing radiation dose. This phenomenon can be attributed to the radiation-induced chain scission process. A similar thermal properties was exhibited by samples treated with the alcohol solutions. In that case the crystallinity degree increased for about 3%. The TPS/PBS blend after sterilization in the autoclave was characterized by slightly lower T_m_ and melt crystallization temperature reduced by ca. 10 °C. The reduction in the T_c_ can be associated with the degradation process as well as to the presence of water interacting with the starch molecules after sterilization and it correlated well with the drop in the T_g_ value.

**Fig. 2:**
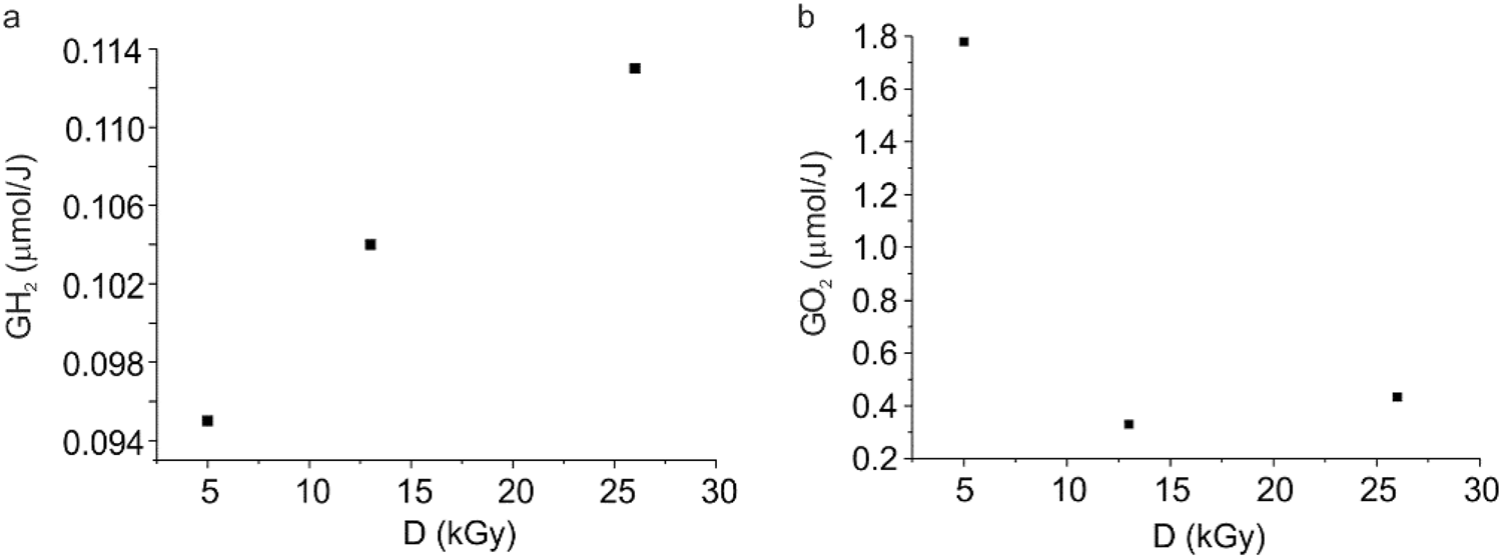
Radiation yield of **a** hydrogen (GH_2_) and **b** oxygen (GO_2_) in the function of radiation dose

In general, during the e-beam irradiation two processes occur: cross-linking and chain scission. Depending on the polymer blend composition, one of those processes can dominate in the system. From the DSC results, it seems that the TPS/PBS blend underwent slow degradation and the creation of the cross-linked structures was limited. The occurrence of the radiation-related cross-linking processes has been confirmed by a gas chromatography on the basis of the hydrogen radiation yield (GH_2_) (Fig. 2a), which is proportional to the number of free radicals [36]. For the analyzed samples, GH_2_ was equal to 0.10 µmol J^−1^. Interestingly, the calculated yield is more than twice lower than in the case of potato starch (GH_2_ = 0.26 µmol J^−1^) and similar to the blend of thermoplastic starch with an aliphatic-aromatic polyester, which exhibit a protective effect against the radiolysis. The cross-linking process was also confirmed by the changes in weight–average molar mass M_w_, which increased with radiation dose (Table 3; Fig. S2). It was well visible especially at the highest dose applied. On the other hand due to the degradation process, which occurred simultaneously, the number-average molar mass M_n_ decreased slightly for the analyzed samples, and as a consequence the increase in molar mass distribution was observed. Taking under consideration the impact of radiation on M_w_ and M_n_ one could expect that cross-linking process is predominant in case of methylene chloride soluble part of studied materials.

**Table 3:** Molar mass and polydispersity of the PBS fraction in the TPS/PBS blend before and after irradiation

Sample	M_n_^a^ (kg mol^−1^)	M_w_^a^ (kg mol^−1^)	Đ_M_^a,b^
0 kGy	11.0	69.5	6.32
5 kGy	10.3	70.8	6.86
13 kGy	10.3	71.1	6.87
26 kGy	10.0	74.8	7.46

^a^Results obtained from refractive index detector based on polystyrene narrow standards calibration

^b^Đ_M_ = M_w_/M_n_

The cross-linking induced by radiation is a well-known feature for PBS and starch. During irradiation of polysaccharides in the presence of oxygen the radicals located on the carbon atoms (C_1_) of glucose units easily react with oxygen forming peroxide radicals. Those radicals can be quickly converted to the ketone group with the elimination of hydroperoxide radical (HO_2_^·^), which in many cases prevents further degradation of polysaccharide chain. This mechanism is unique for polysaccharides, and not observed for any other group of polymers. The influence of glycerol and urea cannot be excluded since they can form hydrogen bonds with starch thus changing its susceptibility to the degradation/cross-linking process [37]. Moreover, it was proved that the presence of such additives as glycerol, isopropanol and *t*-butanol exerts a protective effect by quenching ^·^OH radicals created during water radiolysis and forming the carbon-centered free radicals exhibiting lower reactivity toward polysaccharides [38].

Figure 2b presents the radiation yield of oxygen (GO_2_) in the function of radiation dose. The observed oxygen loss is related to the radiation-induced oxidation of the material and with a higher radiation dose, a lower oxygen diffusion into the polymer is detected [39]. Additionally, irradiation with the dose of 13 kGy or 26 kGy causes the consumption of all available oxygen, thus intensifying cross-linking processes, which is inhabited in the presence of oxygen due to its competition with the oxidative degradation.

**Fig. 3:**
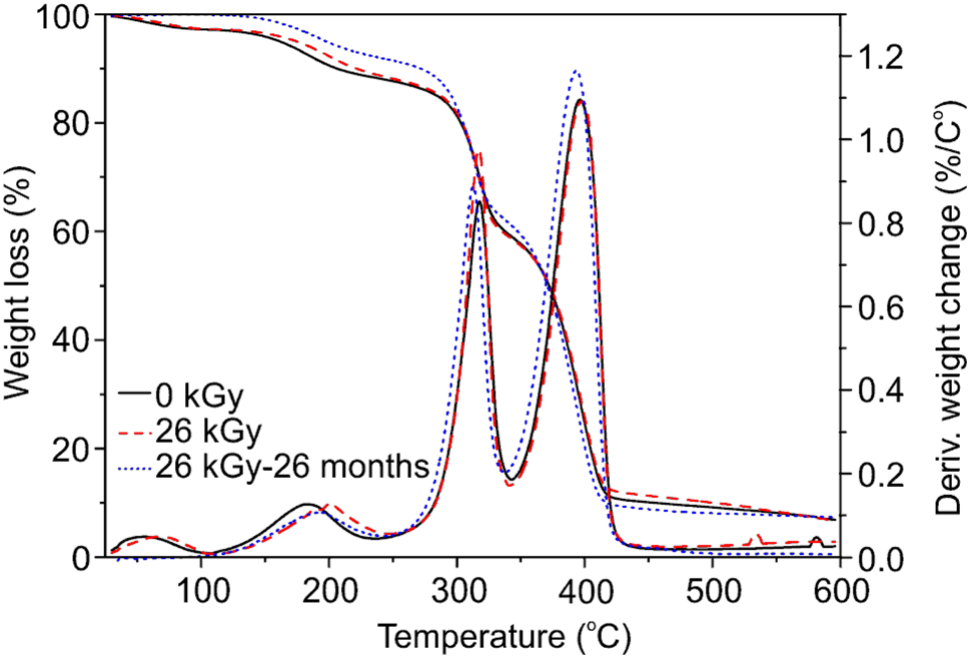
Weight loss and derivatives weight change of the TPS/PBS sample before (black, solid line) and after the e-beam irradiation with a dose of 26 kGy (red, dash line) determined by TG analysis. The blue, dotted line represents the same irradiated sample stored for 26 months (Color figure online)

Figure 3 shows the results from the TG analysis performed for the samples sterilized by the e-beam irradiation. It can be seen that the thermal degradation of each sample underwent through four weight loss stages. The first one up to 120 °C can be assigned to the evaporation of water, and other volatile compounds present in TPS. The weight loss observed between 120 and 230 °C is attributed to the loss of urea and glycerol, which were used as plasticizers [40]. According to the data presented in Table 2, the content of water and plasticizers was stable for all the analyzed samples and reached about 10 wt%. The third stage of thermal degradation relates to the starch decomposition, which began around 250 °C up to 340 °C. The last degradation stage occurred from 350 to 450 °C and can be explained by the PBS chain degradation. Above 450 °C the carbonization process took place.

Based on the collected data, one can conclude, that the T_onset_ of TPS/PBS blend after e-beam irradiation practically did not change, which indicates that probably the chain scission occurred at a very low level or its effect was partially compensated by cross-linking. A very small decrease in thermal stability of the analyzed samples could be observed after 26 months of storage in air. It is especially evident for the sample irradiated with the highest dose. Moreover, it seems that after 26 months a part of the plasticizers evaporated (after its migration towards the surface) since the first weight loss stage did not occur. To summarize the thermal properties of the TPS/PBS blend treated with e-beam radiation, we can conclude that during sterilization process chain scission occurred, tough there might have been also some cross-link in the polymer chain. In general, material after sterilization was characterized by a higher crystallinity degree and unchanged thermal stability, however prolonged exposure to air may deteriorated its thermal properties.

### Mechanical and Barrier Properties Against Water

**Fig. 4:**
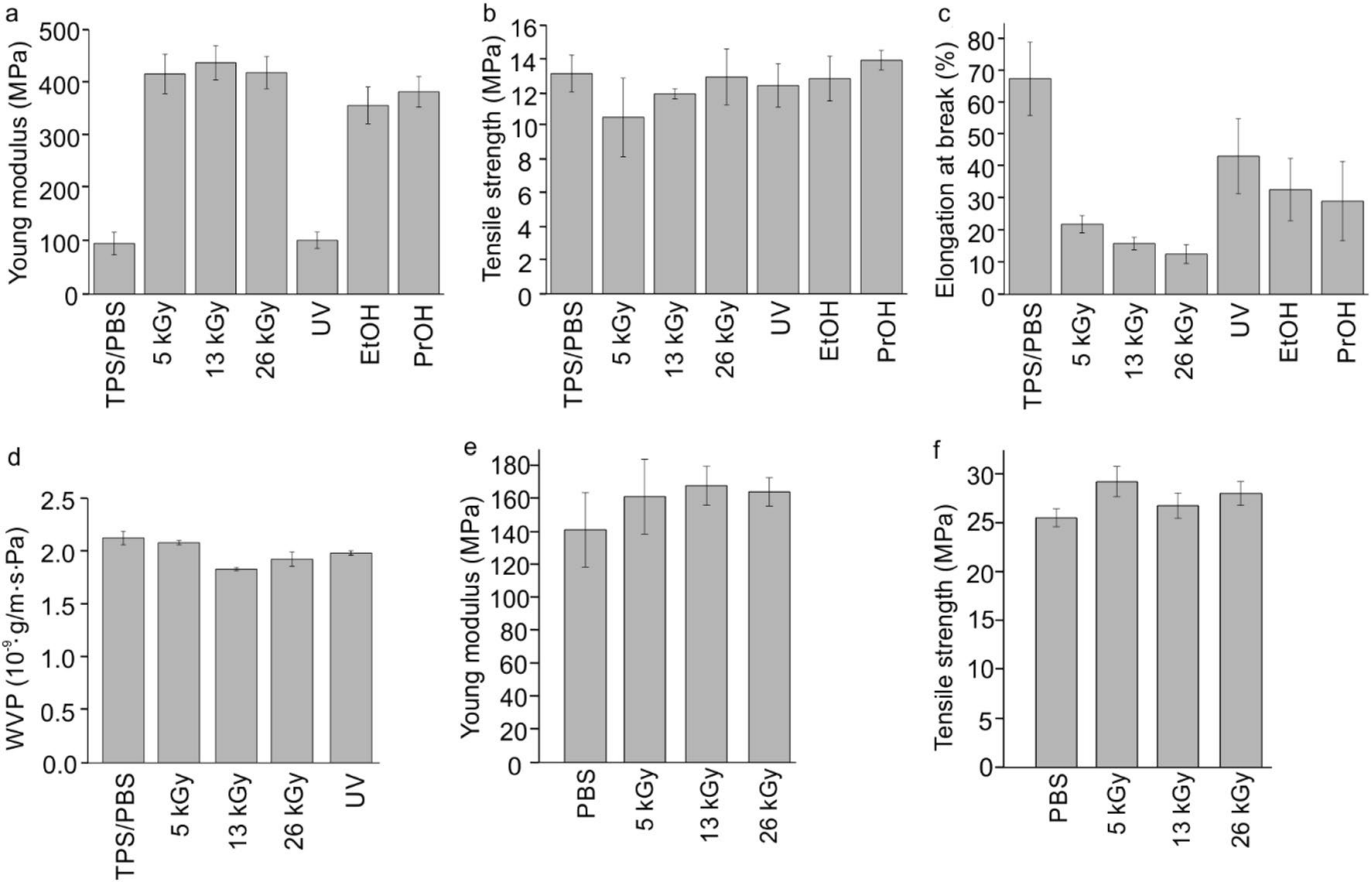
Mechanical properties (**a–c**) and water vapor permeability (WVP, **d**) of the TPS/PBS blend before and after sterilization, and **e**, **f** mechanical properties of PBS before and after irradiation with different doses of the e-beam

The e-beam radiation had a strong impact on the mechanical properties of the analyzed samples (Fig. 4a–c). For all the applied dosages, Young’s modulus of the tested films significantly increased. The polymer became more rigid and was characterized by lower tensile strength and elongation at break than the control sample. The changes in the blend structure had a positive effect on its barrier properties, since the irradiation doses higher than 5 kGy resulted in a reduction of water vapor permeability by about 14% and 10%, respectively (Fig. 4d). Additionally, we tested the mechanical parameters of neat PBS before and after irradiation with the e-beam (Fig. 4e, f) to investigate whether the observed reduction in tensile strength is related to the starch or PBS degradation. As can be seen in Fig. 4e, f the mechanical properties of PBS did not deteriorate after irradiation—they remained the same or even got slightly better compared to the reference sample. In that case, the cross-linking process occurred, however, according to the literature, a stronger cross-linking effect requires higher radiation doses [41]. One can conclude, that in general the e-beam radiation reduces the tensile strength of the TPS/PBS blend mainly due to the chain scission of starch. However it seems that the lower doses of the e-beam irradiation may have stronger effect on degradation than the higher ones. Other sterilization methods, performed in the presence of ethanol and isopropanol resulted in an increase of Young’s modulus by almost 3.5 times in comparison to the control sample. This effect was caused mainly by leaching of plasticizer to the alcohol solution. In that case the material became less elastic and the reduction of elongation at break was observed. It seems that only the UV radiation did not change significantly the mechanical parameters of the TPS/PBS blend, and additionally slightly enhanced its barrier properties against water.

### Morphology of the Blends

**Fig. 5:**
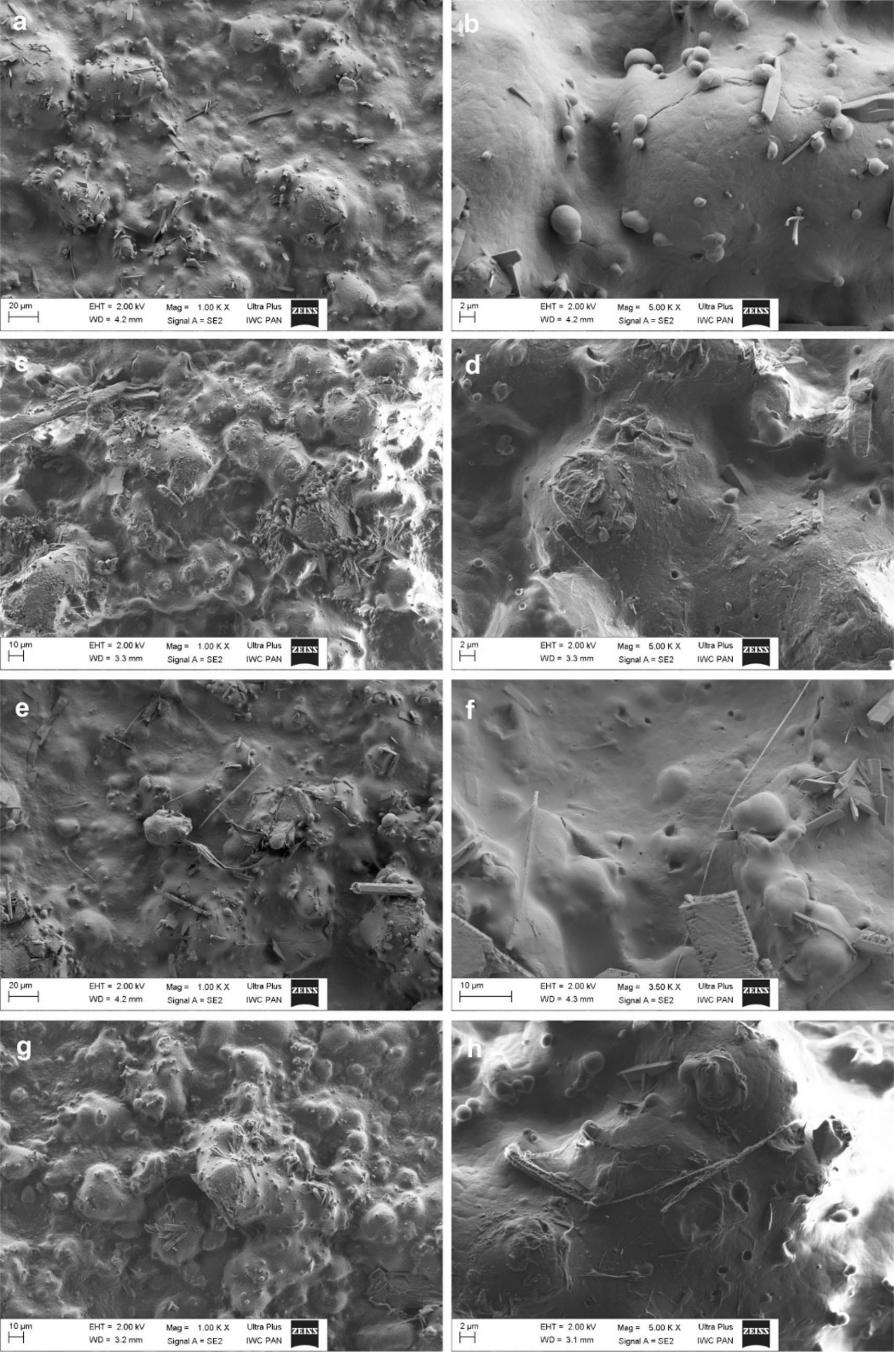
SEM images of the neat TPS/PBS blend (**a**, **b**) and its samples irradiated with different doses of the e-beam: 5 kGy (**c**, **d**), 13 kGy (**e**, **f**) and 26 kGy (**g**, **h**)

The morphology of the blend strongly influence its mechanical properties, therefore a microstructure of the TPS/PBS samples was analyzed by SEM. Figure 5 shows the SEM microphotographs of a non-irradiated TPS/PBS and its samples treated with the e-beam. As shown in Fig. 5a, b, starch and PBS created two immiscible phases. The blend exhibited a matrix-dispersed phase morphology, particles of starch retain their granular shape, and are dispersed in the PBS continuous matrix [42]. The difference in a polarity between a polyester and a polysaccharide causes difficulties in obtaining the miscible system [43]. Moreover, on the surface some tetragonal crystals can be observed—it is possible that they are formed by urea. In Fig. 5b, we can also observe small particles located on the polymer surface. We suspect that they can be made of the crystalline starch since the similar structures of starch were already observed by several authors [44]. After irradiation with the e-beam (Fig. 5c–h) morphology of the blend did not change significantly, however, in some regions of the sample, the small cavities/holes could be observed. It should be mentioned, that the number of those holes did not change an increase of the radiation dose. These results suggest that the radiation caused a partial damage to the blend surface, however, the polymer phase integrity has been maintained.

### Biodegradation Studies

Starch degradation occurs as a consequence of enzymatic attack at the glycosidic linkages between the glucose units. The main enzymes responsible for that process are amylolytic enzymes such as: α- and β-amylase, glucoamylase, isoamylase, pullulanase, exo-1,4-α-d-glucanase, α-d-glycosidase as well as cyclomaltodextrin-d-glucotransferase [45]. In our studies we examined the degradation rate of the irradiated TPS/PBS blend in the presence of a commercial α-amylase and crude amylase synthesized in *B. subtilis* cultures. For industrial application the purification of α-amylase is not required, however, for the pharmaceutical and biomedical purposes such process is obligatory [46]. For that reason, in our work we used both types of this degradation agents: pure α-amylase and lyophilizate of the post-culture liquid from *B. subtilis*.

#### Degradation in the Presence of a Commercial α-Amylase

**Fig. 6:**
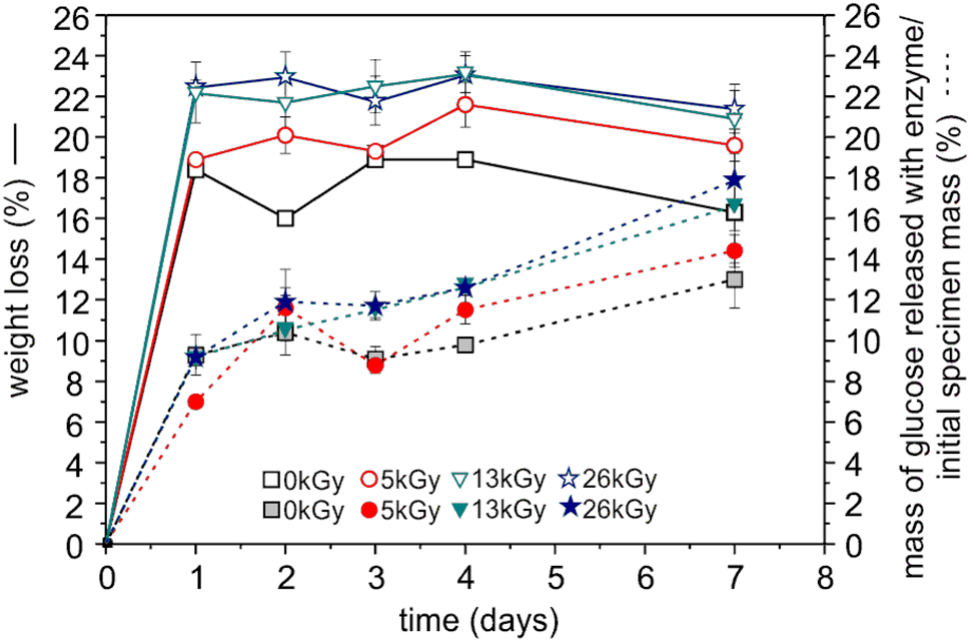
Weight loss (solid line and empty marker) and mass of glucose released to the solution (dashed line and filled marker) during the TPS/PBS blend degradation, as a function of degradation time

The simplest method to investigate the changes occurring during polymer degradation is the gravimetric method, where the sample is weighed before and after degradation and a percentage of the weight loss is calculated. For TPS and its blends the results obtained from this method may be overstated due to the high percentage of water and plasticizers which they typically contain. That is why, in the presented studies we showed the degree of degradation of the TPS/PBS blend before and after radiation, expressed as weight loss and mass of glucose released to the degradation solution. As can be seen from Fig. 6, it seems that the irradiation caused an acceleration of the sample degradation, regardless of the adopted evaluation criterion. Probably, the small holes observed in the SEM images of the irradiated samples, facilitated a penetration of water into the sample thus making a degradation of starch more effective. Those results are in agreement with the literature [47].

The amount of polysaccharides released to the solution was also determined (Table S1 in Supplementary Material), by a previously described colorimetric method [24]. It was found that the quantity of starch released in all degradation experiments was small and not exceeded 0.30%. This finding allows for a conclusion that the estimated weight loss was associated mainly with the release of glucose and plasticizers. Since initially the investigated blend contained 50 wt% of PBS, 35 wt% of starch, 12.5 wt% of urea and 2.5 wt% of glycerol, it seems that after 7 days of degradation, a maximum half of starch amount has been decomposed (18% in case of sample irradiated with a dose of 26 kGy, calculated by the mass of glucose method as shown in Fig. 6) and we assume that the rest of it was still present in the PBS matrix. Probably, this remaining starch phase is strongly interpenetrated with the insoluble PBS component, which makes it difficult for enzymes to reach its molecules.

**Fig. 7:**
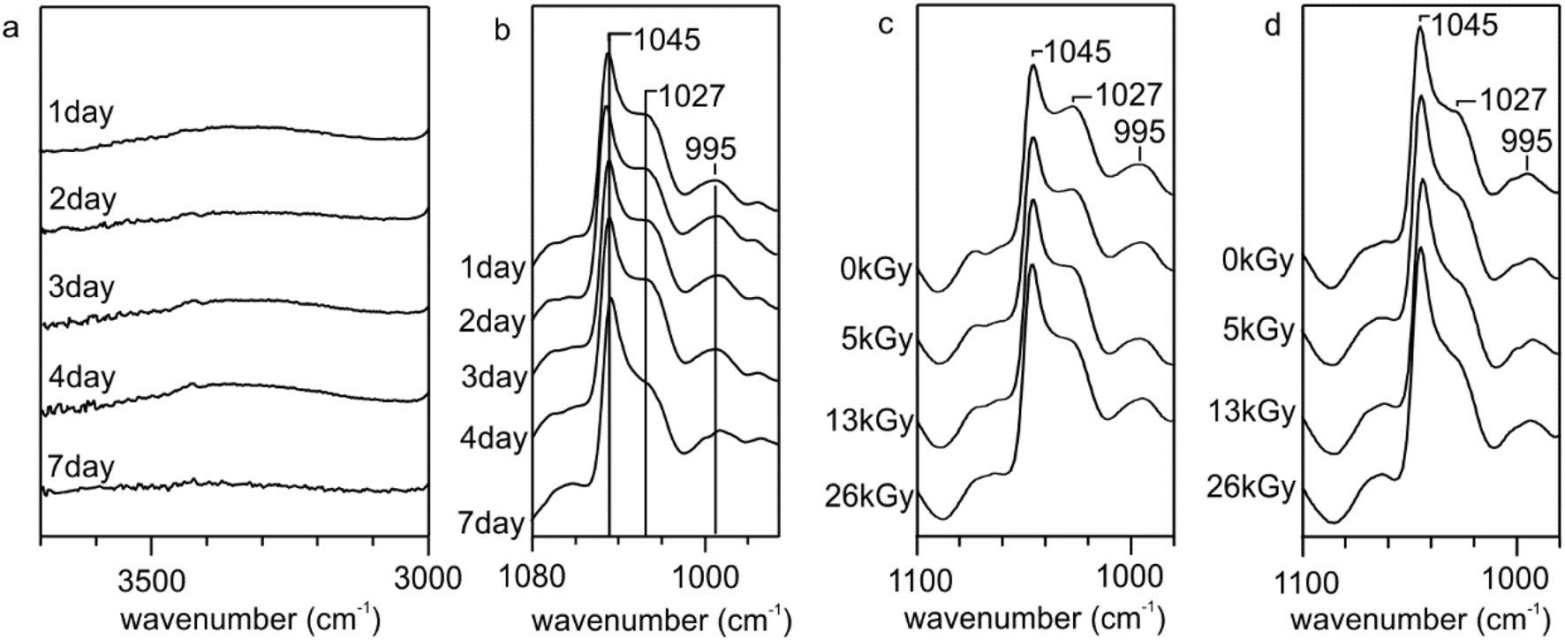
FTIR spectra of the TPS/PBS blend: irradiated with a dose of 13 kGy subjected to degradation, in the region of **a** 3700–3000 cm^−1^ and **b** 1080–960 cm^−1^; non-irradiated and irradiated with different doses of the e-beam, after 1 day (**c**) and 7 days (**d**) of degradation, in the region of 1100–980 cm^−1^

To specify the surface changes of the TPS/PBS blend after degradation, starch infrared absorption bands characteristic of the C–O–C (at 1027 cm^−1^ and 995 cm^−1^ (Fig. 7a) and –OH groups (in the range of 3700–3000 cm^−1^, Fig. 7b) were evaluated. For all the examined samples, regardless of the applied radiation dose (here we present the FTIR spectra only for the samples treated with a dose of 13 kGy), a starch degradation from the polymer surface began immediately, without any prolonged induction period. One can clearly observe that the intensity of the analyzed signals decreased with the degradation time, and after 7 days the signal characteristic of the OH group was barely detected.

Moreover, as can be seen in Fig. 7c, d, at the beginning an increase in the applied e-beam dose accelerated starch degradation, however, after 7 days of incubation this phenomenon was negligible. Those observations were also supported by the SEM pictures (Fig. S3 in Supplementary Material), which showed that the changes in the surface topography of the samples non-irradiated and irradiated with a dose of 26 kGy after 7 days of enzyme action were similar. Both analyzed films had small holes on their surface which arise from a degradation of starch.

**Fig. 8:**
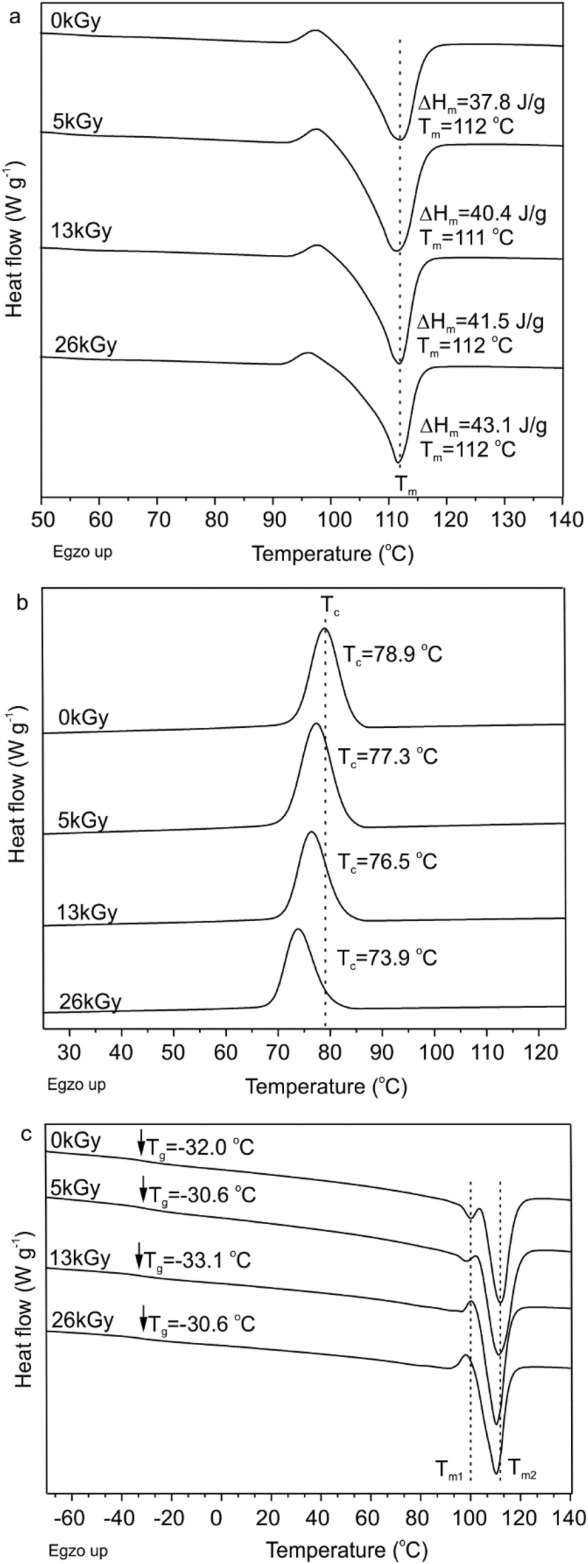
DSC thermograms of the TPS/PBS blends after 7 days of enzymatic degradation: **a** the first heating cycle, **b** the cooling cycle, and **c** the second heating cycle. T_m_, T_c_ and ΔH_m_ refer to the melting temperatures, melt crystallization temperature and enthalpy of melting, respectively

After 7 days of enzymatic degradation all samples were tested by means of DSC. Melting temperature remained unchanged, however its enthalpy of melting was gradually increased with the increasing radiation dose (Fig. 8a). This was expected due to the fact, that during hydrolysis the amorphous phase of aliphatic polyesters degrades more rapidly than the crystalline phase [48]. The radiation-induced chain scission of PBS had also an effect on the melt crystallization temperature (Fig. 8b), which was shifted to the lower region when the radiation dose increased. One can expect that, the oligomers formed during degradation, hindered crystallization of the PBS. According to the literature, the polymer with lower molecular weight exhibits much lower T_c_ and the crystallization is much slower. Moreover, the nucleation density increases with increasing molecular weight [49]. In our case biodegradation and radiation-induced chain scission probably caused lowering of the PBS molecular weight and as a consequence we observed a decrease in T_c_. Interestingly, a double peak from melt-recrystallization process of PBS (Fig. 8c) appeared only for the non-irradiated sample and that subjected to the lowest radiation dose. For the rest of the samples the first melting peak (assigned as T_m1_ in the Fig. 8c) was not detected, which suggest that the melt-recrystallization process did not occur. Instead, a cold crystallization can be observed, as evidence by the occurrence of a small exotherm (T = 100 and 98 °C for the samples irradiated with 13 and 26 kGy, respectively) just before the melting peak.

#### Degradation in the Presence of Crude Amylase Synthesized in *B. subtilis* Cultures

The *Bacillus* species are considered as the most important producers of α-amylase and are widely used in the industry, for example in the solid state fermentation [50]. Except of starch degradation, some *Bacillus* species like *B. pumilus* and *B. subtilis* can also be active in the aliphatic polyester decomposition, because they are present in their degrading microbiota in natural environments under mesophilic conditions [51].

The α-amylase production by *B. subtilis* bacteria was conducted in a culture medium containing 2% of soluble starch, yeast extract and peptone. The optimum temperature and time of incubation was 37 °C and 72 h, respectively (Fig. S4 in Supplementary Material). The post-culture liquid (supernatant), with the highest amylolytic activity (0.27 U mL^−1^) was used as a degrading agent for the blend subjected to the e-beam radiation. The αamylase produced by *B. subtilis* cells showed the highest activity at 50 °C, however, decreased rapidly with time and even after 1 h of incubation 10% of that activity was lost. Thus the polymer degradation was conducted at slightly lower temperature of 37 °C.

**Fig. 9:**
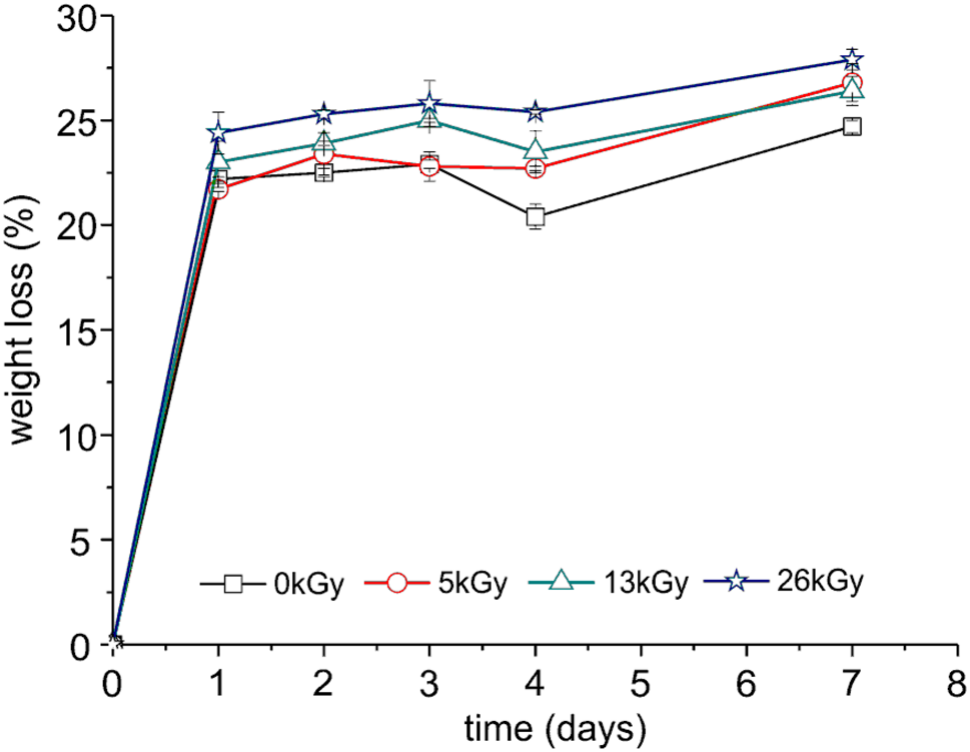
Weight loss of the studied TPS/PBS blend before and after irradiation by different doses of the e-beam as a function of degradation time in the presence of crude amylase

As can be seen in Fig. 9, the investigated polymer blends underwent fast degradation in the first 24 h since in that timespan the weight loss measured for all samples exceeded 20%. It seems, that a decomposition of the sample irradiated with the highest dose of the e-beam was the fastest and after 7 days of incubation it lost 28% of its initial weight. Moreover, these results shows, that the crude amylase from *B. subtilis* cultivation was more efficient in degradation of the TPS/PBS blend than pure α-amylase, even if its activity was much lower. The post-culture liquid is a mixture of different isoenzymes of amylases, which are able to hydrolyze starch in different chain position. On the other hand, α-amylase is responsible only for decomposition of the α-1,4-glycosidic linkages. This can explain, why using lower-purity enzyme resulted in a higher degradation degree.

DSC analysis (Table 4) of the samples after degradation with crude amylase showed an increase of the glass temperature at higher radiation doses, which is indicative of a partial degradation of the PBS amorphous phase. This effect can be also related to the leaching of plasticizers, however, such a rise of the T_g_ value was not observed after degradation with α-amylase. For all samples degraded with a crude amylase, the enthalpy of fusion was higher in comparison to the undegraded sample by approximately 11 J g^−1^. The most significant changes were observed in the melt crystallization temperature, which decreased with an increasing radiation dose. The same effect was also observed during experiments with α-amylase and can be explained in the same way by the creation of PBS oligomers during degradation.

**Table 4:** Thermal properties of the non-irradiated and irradiated TPS/PBS blend after 7 days of degradation in the presence of crude amylase

Sample	T_g_ (°C)	T_m_ (°C)	ΔH_m_ (J g^−1^)	T_c_ (°C)
0 kGy	− 33.5	112	43.0	80.5
5 kGy	− 31.8	112	40.8	78.1
13 kGy	− 26.7	111	41.5	76.8
26 kGy	− 29.7	112	43.4	74.4

T_g_ and T_m_ were determined from the 2nd and 1st heating scan, respectively; T_c_ was determined based on the cooling cycle. T_g_, T_m_, T_c_ and ΔH_m_ refer to the glass temperature, melting temperature, melt crystallization temperature and enthalpy of melting, respectively

In our studies we evaluated a degradability of the TPS/PBS blend under the influence of *B. subtilis* cells. Such experiments were performed on the Petri dishes filled with mineral medium (nutrient-salts agar, without any additional carbon source). The weight loss, thermal and structural properties were examined for each material after 1, 2 and 3 weeks of incubation at 37 °C (Table 5). This variant of degradation forces microorganism to use the TPS/PBS blend components as their only source of carbon for cell proliferation.

**Table 5:** Thermal properties of the TPS/PBS after 1, 2 and 3 weeks of degradation on the Petri dish in the presence of *B. subtilis* cells

Degradation time (weeks)	Weight loss (%)	T_g_ (°C)	T_m_ (°C)	ΔH_m_ (J g^−1^)	T_c_ (°C)	T_onset3_ (°C)	T_onset4_ (°C)
1	26.6 ± 3.3	− 26.3	111	41.0	78.1	298	373
2	27.8 ± 0.5	− 33.3	111	44.5	78.7	–	–
3	40.9 ± 3.1	− 29.3	112	53.4	81.5	280	370

T_g_ (glass transition), T_m_ (melting temperature) and ΔH_m_ (enthalpy of melting) were determined by the 2nd and 1st heating scan, respectively. T_c_ (melt crystallization temperature) was determined by cooling scan.; T_onset3_ and T_onset4_ relate to the degradation of TPS and PBS in the polymer blend, respectively

**Fig. 10:**
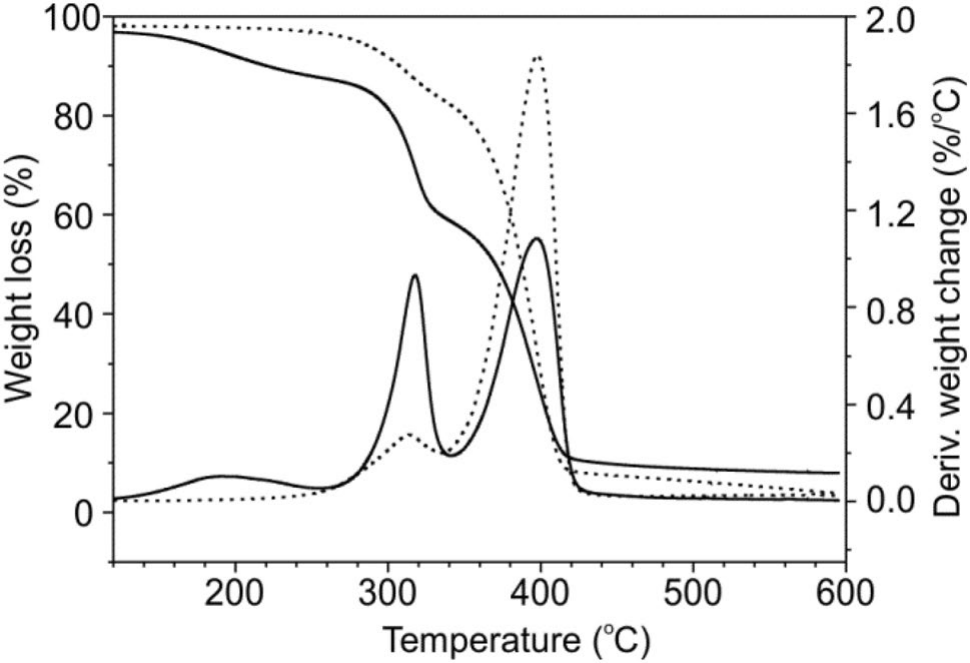
TG analysis of the TPS/PBS blend before (solid line) and after (dashed line) 3 weeks of degradation in the presence of *B. subtilis* cells on the agar plate

Similarly to the results obtained with amylase enzyme, after 1 week of degradation about 26.6% of the blend was degraded. During the second week the weight loss of the sample did not change significantly, however longer degradation increased its value to almost 41%. The melting temperature of the PBS crystalline phase was stable and close to T_m_ of the non-degraded sample. Moreover, during the incubation, the ΔH_m_ value was gradually increased which confirms a degradation of the PBS amorphous phase and is in a good agreement with the results obtained during a liquid degradation. The melt crystallization temperature during cooling increased to 78–81 °C, which can be explained by the presence of oligomers and their greater mobility and ease of rearrangement into crystal domains. From the TG thermogram (Fig. 10) one can see that after 3 weeks of degradation the sample contained mainly the PBS phase and the fraction of starch was much smaller than at the beginning of the experiment—this finding is supported by the weight loss around 300 °C getting smaller after degradation.

## Conclusion

The effect of the e-beam radiation and other commonly used sterilization techniques on the structural, morphological, and biodegradation properties of the TPS/PBS blend has been explored. It turned out that all the applied sterilization methods effectively eliminated the growth of yeast and molds, while only the e-beam irradiation decreased the bacterial microflora below 1.0 log cfu mL^−1 ^. In addition, according to the SEM and FTIR analyses, the applied e-beam radiation doses did not cause significant changes on the surface of the investigated material. On the other hand, they improved barrier properties of the material against water vapor and allowed faster decomposition in the presence of a commercial α-amylase and enzymes of microbial origin (derived from wild strain *B. subtilis*). This means that during the irradiation both degradation and cross-linking processes took place, which additionally has been confirmed by a gas chromatography on the basis of the hydrogen radiation yield. All the analyzed sterilization methods worsened the mechanical properties of the tested material, since the polymer blend became more rigid and less flexible, however, the UV sterilization had the lowest impact on the mechanical parameters. The obtained results revealed that among all the studied techniques, the e-beam irradiation is the most promising for sterilization of the TPS/PBS blend due to its high efficiency in microorganism elimination, positive effect on both the barrier properties against water and degradation of the blend. The other methods, which did not give such good results, will be the subject of further research, where a combination of different techniques will be studied.

## Electronic supplementary material

Below is the link to the electronic supplementary material.

(DOCX 247 kb)
