# Supplementary material for: Effect of Electron-Beam Radiation and Other Sterilization Techniques on Structural, Mechanical and Microbiological Properties of Thermoplastic Starch Blend
Source: J Polym Environ. 2020 Nov 21;29(5):1489–504. doi: 10.1007/s10924-020-01972-9 (PMC7679798; doi:10.1007/s10924-020-01972-9)
Supplement: Supplementary file 1 — (DOCX 247 kb) [file 10924_2020_1972_MOESM1_ESM.docx]

**Supplementary material**

**Effect of electron-beam radiation and other sterilization techniques on structural, mechanical and microbiological properties of thermoplastic starch blend**

**Authors:** Anna Iuliano^1,2*^, Agata Fabiszewska^1^, Katarzyna Kozik^1^, Magdalena Rzepna^3^, Justyna Ostrowska^4^, Maciej Dębowski^2^, Andrzej Plichta^2^

**Affiliations:**

^1^Department of Chemistry, Institute of Food Sciences, Warsaw University of Life Sciences – SGGW, Nowoursynowska 159c, 02-776 Warsaw, Poland

^2^Faculty of Chemistry, Warsaw University of Technology, Noakowskiego 3, 00-664 Warsaw, Poland, *aiuliano@ch.pw.edu.pl

^3^Institute of Nuclear Chemistry and Technology, Dorodna 16, Warsaw 03-195, Poland

^4^Department of Organic Technologies, The Łukasiewicz Research Network – New Chemical Syntheses Institute, al. Tysiąclecia Państwa Polskiego 13A, 24-110 Puławy, Poland





**Fig. S1.** DSC curves of the TPS/PBS blend before and after sterilization recorded during the 2^nd^ heating carried out at 20 °C min^−1^ rate.





**Fig. S2.** GPC chromatograms of the PBS fraction in the PBS/TPS blend: non-irradiated and irradiated with a dose of 5 kGy (a), 13 kGy (b) and 26 kGy (c).





**Fig. S3.** SEM images of the TPS/PBS surface after 7 days of degradation in the presence of commercial α-amylase: non-irradiated (a) and irradiated with a dose of 26 kGy (b).





**Fig. S4.** Influence of incubation time on the activity of amylase derived from *B. subtilis*.

**Table S1.** The effect of degradation process on the mass of starch released to the degradation solution.

| Time of degradation | Mass of starch  g starch /g specimen (%) | | | |
| --- | --- | --- | --- | --- |
|  | 0 kGy | 5 kGy | 13 kGy | 26 kGy |
| 1 day | 0.24  ± 0.03 | 0.28  ±0.03 | 0.29  ±0.02 | 0.29  ±0.02 |
| 2 day | 0.27  ±0.02 | 0.22  ±0.04 | 0.26  ±0.03 | 0.26  ±0.05 |
| 3 day | 0.14  ± 0.04 | 0.17  ±0.03 | 0.28  ±0.05 | 0.27  ±0.02 |
| 4 day | 0.13  ± 0.01 | 0.27  ± 0.04 | 0.18  ± 0.02 | 0.20  ± 0.02 |
| 7 day | 0.15  ±0.01 | 0.19  ±0.01 | 0.22  ±0.03 | 0.21  ±0.06 |
